# The Spherical Tokamak for Energy Production (STEP) in context: UK public sector approach to fusion energy

**DOI:** 10.1098/rsta.2023.0401

**Published:** 2024-08-26

**Authors:** Adam Baker

**Affiliations:** ^1^ Department for Energy Security and Net Zero, 3-8 Whitehall Place, London SW1A 2AW, UK

**Keywords:** fusion energy, government policy, public investment, STEP

## Abstract

The UK’s fusion energy approach has developed over the past 5 years to include government policy initiatives and a range of public sector investments designed to be delivered in partnership with the private sector. These have aimed to create an environment that stimulates innovation and investment to deliver economic as well as scientific and environmental benefits throughout the lifetime of the public sector fusion energy programme. The Spherical Tokamak for Energy Production acts as a focus and anchor for both public and private sector efforts to develop fusion energy, developing the supply chain and potential for Intellectual Property development and export opportunities well ahead of the anticipated STEP completion date of 2040. This is maximized by the UK’s approach to a holistic research and innovation programme backed up by a regulatory and skills programme.

This article is part of the theme issue ‘Delivering Fusion Energy – The Spherical Tokamak for Energy Production (STEP)’.

## 1. Introduction

The Spherical Tokamak for Energy Production, or STEP, was announced by the UK Government in 2019 [1]. It promises a programme of work to build a prototype fusion power station by 2040 that would be capable of putting energy on the grid; £220 m was set aside for its concept design. The media has reported extensively on the programme [2,3] and STEP has become a visible totem for the UK fusion energy programme, offering a route to low carbon, safe and virtually inexhaustible energy.

However, this only tells part of the story of the UK’s approach to developing fusion energy. Over the last 40 years, the UK has developed world-leading and globally recognized expertise in fusion R&D. This comes from experience of running the Joint European Torus (JET), for many years the most advanced fusion reactor in the world, and other cutting-edge facilities. But the UK’s progress in this field also comes from the institutional strength and focus of UK Atomic Energy Authority (UKAEA) and the supportive policy and regulatory environment put in place by government.

STEP sits within a much broader set of government interventions designed not just to develop technical solutions to fusion energy generation, but the commercialization of the technology and to create a vibrant fusion energy sector in the UK capable of competing on the global stage [4]. The benefits of fusion energy are not just environmental: the economic benefits could be considerable [5,6] if the UK maintains a competitive edge in the global race for fusion. This paper will set out that wider UK approach and show how STEP fits into, contributes to and helps move UK fusion energy forward.

## 2. Strategy drivers

The key assumption driving the UK’s strategy is that the UK can and should lead the world in the development of commercial fusion energy, and export technology and expertise into a global green energy market, bringing significant economic and environmental benefits. With global demand for energy expected to continue growing [7], this is a long-term goal aimed squarely at achieving and maintaining *global* net zero emissions and capturing the economic returns of that, rather than being a major contributor to the UK’s own net zero targets. Nonetheless, UK interventions anticipate economic benefits in the meantime. While the magnitude of benefits is yet to be fully understood, it is likely that the creation of high-skilled jobs, commercial and development opportunities for the UK’s fusion supply chain, secondary technology transfer opportunities and development of a robust skills pipeline will result in significant positive externalities well before STEP begins operation. With that in mind, the UK currently has two mutually reinforcing objectives:

- To build by 2040 a prototype fusion power station (STEP) capable of putting energy on the grid.
- To develop a vibrant fusion energy sector in the UK.

The first of these objectives provides a driver for the second, creating commercial opportunities and reducing the financial risk for private sector bodies that want to develop fusion capability; in essence, STEP helps create a lower risk market in which to gain a foothold. Critically, it also drives technology development and research, creating a focus for private and public sector efforts in fusion, efforts which the UK envisages will be of benefit to more than just the spherical tokamak approach to fusion. Necessary developments in advanced materials, tritium breeding and handling, remote maintenance and heat extraction are of value to most fusion technologies and a supply chain capable of providing those solutions would be capable of servicing a far more diverse range of fusion technologies. Given the expertise of the UKAEA in spherical tokamaks, it is expedient and prudent to use that approach for a prototype fusion power plant, but the UK fusion approach as a whole is technology agnostic, with a focus (outside of the STEP project) on broad application technology development and sector support.

The second objective, development of a vibrant UK fusion energy sector, skilled and capable enough to supply both the STEP and a global fusion market is a pre-requisite for the STEP project and ensures that the economic benefits of building STEP rightly remain (as far as possible) in the UK, given the level of UK government financial support. It is this objective that will ultimately lead to the greatest long-term economic benefit post-STEP as sector capability is grown and consequently export opportunities increase.

This sector development activity recognizes that magnetic confinement fusion is not the only approach to fusion energy generation and that the UK must be ready to compete in a rapidly evolving sector. Inertial confinement fusion has had significant recognition lately given results at the National Ignition Facility in the United States [8] and a wide range of other approaches are being developed worldwide including stellarators, accelerator-based fusion and hybrid (fission/fusion) machines. The UK’s approach to dealing with this diversity is to support sector capability development in technologies that support common elements of commercial fusion and are widely applicable. Whilst STEP is envisaged as a market accelerator, the support for supply chain development that is broader helps drive, and somewhat buffer, UK fusion sector competitiveness as multiple technological approaches to fusion develop.

## 3. Public sector interventions

There are three policy workstreams being driven in the UK public sector that, together, describe the interventions taken to achieve UK fusion objectives. These are described as science and technical leadership, international leadership and commercial leadership. Although a convenient way of describing policy, it should be noted that these workstreams are interdependent and reinforcing, no one intervention is entirely described by just one of these labels. These were originally set out in Towards Fusion Energy: The UK Fusion Strategy in 2021 [9] and updated in 2023 [4].

## 4. Science and technology

This workstream is primarily focused on further developing UK R&D capability in fusion, building on the expertise developed in UKAEA over the last 40 years and ensuring that the UK remains at the forefront of fusion science. STEP is the most obvious example of this approach, acting as an anchor project and focus for research efforts into its constituent parts (e.g. plasma physics, advanced materials, tritium breeding). STEP will be delivered by UK Industrial Fusion Solutions (UKIFS), a limited company set up by government, a vehicle better suited to developing the commercial relationships and expertise required to deliver a project of this scale than a purely public sector body. While initially incubated in UKAEA, the intention is that STEP will eventually become independent. To support UKIFS, UKAEA will act as their science partner providing the R&D capability needed to overcome the technical barriers needed to deliver it, and organized along lines that directly address each technical barrier it faces.

Beyond STEP, the Culham Campus in Oxfordshire brings together a range of facilities each focused on a different technical challenge that must be addressed to deliver commercially viable fusion energy. The MAST-U machine is testing how to exhaust heat from a spherical tokamak, a key challenge for a more compact machine. The super-X divertor has already shown that heat exhaust can be successfully managed [10] and providing confirmation that computer modelling used to predict results is robust and accurate. Other facilities include those looking at remote access to challenging environments, necessary to reducing downtime in commercial fusion machines that will need high availability to be commercially successful. The Materials Research Facility is looking at advanced materials (and their characterization) necessary to ensure a commercially viable lifetime for fusion machines, as well as how to handle the modest amounts of intermediate-level waste generated in fusion machines.

One of the most recent additions to the campus is the Hydrogen 3 Advanced Technology (H3AT) centre, announced as part of the fusion foundations programme in 2020 [11]. This facility is designed to research tritium and its processing, storage and disposal, the first custom-built facility of its kind and essential in helping develop the fuel handling capability required by STEP and other fusion energy technologies. This capability will be enhanced by a new facility to research the fusion fuel cycle, the Lithium Breeding Tritium Innovation (LIBRTI) facility [4]. This new facility will work closely with H3AT to tackle currently unanswered questions around how tritium breeding in fusion machines will work, in this case, using lithium. A capacity essential to maintain fusion generation in STEP, tokamaks and other fusion technologies that use tritium. Other programmes look at artificial intelligence-supported design, systems integration, decommissioning and, crucially, skills.

Alongside these newer facilities sits JET. A cornerstone of UKAEA and EU fusion science for decades. While JET performed its last world record beating [12] fusion experiments at the end of 2023, it has reached a new and equally important stage of its lifecycle. JET will be the first D–T-based fusion machine to be decommissioned, an exercise that will in itself provide critical R&D necessary to understand the most effective and efficient methods of decommissioning, as well as the costs and techniques involved, essential for the development of costings for future power stations. While not all these facilities, and others like them on the Culham campus are unique, their co-location provides a currently unique opportunity to bring those technologies and experts together, with STEP as an anchor (but not only) customer, better allowing exchange of ideas, mutual understanding and in the end, system integration. STEP provides an impetus for the development of necessary capability, and STEP is reliant on those capabilities being developed, they are interdependent.

In essence, the science and technology strand to ensure that the UK retains a cutting-edge fusion R&D capability, and maximizes the impact of those capabilities using co-location, coordination and cooperation through a central authority, is the UK Atomic Energy Authority. Without that central, expert, strategic coordination role, there is a danger that the goals of each technology strand diverge. UKAEA and STEP unify those efforts.

## 5. International collaboration

The value of international collaboration in science is well known [13], and this holds just as true for fusion, perhaps more so, given the significant capital costs involved in building fusion machines. The UK approach acknowledges this and aims to help accelerate global fusion development through use of international collaborations but is also realistic in that it aims to strike a balance between collaboration and protecting specific IP, ensuring that there is a decent return on considerable public sector investment. This balanced approach to international collaboration is essential if a case for public investment in STEP and other fusion energy R&D is to be made. This pragmatism sits at the heart of the strategy’s international collaboration approach. The biggest change in the UK’s approach to international collaboration in the last few years was the decision not to associate to the Euratom Research and Training Programme and instead launch a £650 m programme called Fusion Futures [4], driven largely by the decrease in value for money of UK association in the years after the UK–EU Trade and Cooperation Agreement was concluded. It is important to note that this decision did not preclude ongoing collaboration with ITER through a different route; the UK Government at the time was clear [14] that it wants ITER to succeed and ongoing UK–ITER collaboration is mutually beneficial, despite press speculation to the contrary [15]. That said, a decision was taken that the UK’s own Fusion Futures programme was better value for money than full association to the Euratom Research and Training programme, and the stronger focus on globally valuable capabilities such as the new LIBRTI facility, supply chain development, skills and a wider set of global collaborations was the best way forward. It is interesting that the existence of a fully developed STEP programme gave the UK more options when deciding whether association was the best option for the UK, even if it was not the deciding factor.

It is worth noting that most of the funding earmarked for Fusion Futures (over 60%) is intended to go to the private sector, through contracts to build LIBRTI and other components, and the UK’s fusion industry programme that co-funds private sector R&D programmes. The fusion futures programme also provides for a future relationship with ITER, as well as putting funding aside (£25 m) for new international collaborations should negotiations prove fruitful [16].

The UK has already signed two new collaboration agreements with the United States of America [17] and Canada [18] and is in discussions with other potential partner countries. These new agreements envisage collaboration in areas such as skills, public perception of fusion and regulation and lay the groundwork for ensuring that our respective fusion programmes are mutually supportive and make the most of the capabilities and technology we share.

The collaboration opportunities inherent in a global fusion landscape that has seen public investment in fusion grow considerably are obvious, and a key plank of UK fusion activity is to be as open as possible to partnership. The UK also has a role to play in multilateral fora such as the IAEA (International Atomic Energy Agency) and the G7 to help drive further cooperation including in areas such as standards and regulation. Given the depth of UK expertise and experience, there is much to add to those wider conversations about fusion’s role in energy security and global warming.

## 6. Commercial leadership

Over the last few years, the focus of fusion endeavour, in the UK and globally, has shifted markedly from a largely R&D-based approach to one that is taking commercialization of fusion technology seriously. As of 2023, there were over 40 private sector fusion companies in the world and private sector investment is estimated at around £7 billion [19], the private sector is moving fast and the public sector, if it is to support this development, must adapt to serve it. In the UK, one of the biggest shifts was to back STEP, an endeavour aimed specially at *commercially viable* fusion, aiming at smaller more cost-effective design than ITER and with a strong focus on maximizing availability as well as performance. It will be a collaboration between the public and private sector, with engineering and construction partners having a key role in engineering design. The premise, as mentioned above, is that STEP acts as a catalyst to drive private sector capability development to the point where regardless of STEP performance, they can take what they have learned and use that to develop new products and export globally, even before STEP is finished.

Since then, other programmes have been implemented to help drive UK private sector development. The Fusion Industry Programme, which has been running since 2022, offers funding to companies and others to jointly develop specific pieces of fusion technology. So far, 91 organizations have benefited from this, and the fusion futures programme will boost funding by an additional £35 m [16]. In addition, many of the cutting-edge facilities and capabilities developed by UKAEA are available for UK-based companies to collaborate with, boosted by efforts to develop a fusion cluster in both Culham and (in time) at the STEP site in Nottinghamshire. The cluster or campus approach to fusion facilities and capability has significant benefits from an industry perspective, as these facilities present a commercial opportunity. By opening the facilities up to collaborate with the supply chain, undertaking joint-funded projects, and sharing learning with partners, these facilities provide a chance for UK-based companies to upskill, providing not just technical solutions, but potential export opportunities based on new IP generated. This in turn incentivizes co-location of private sector companies nearby, creating a fusion cluster that maximizes positive agglomeration impacts by collocating the public sector capabilities and a high concentration of fusion-based private sector organizations, adding to the skills and experience available in the UK as well as enhanced opportunities for tech transfer.

The latter has been enhanced further with a new £18 million tech transfer hub, announced as part of the Fusion Futures programme [16]. This is essential not just for the eventual commercialization of STEP, but the exploitation of the technology developed along the way. Both governments and companies want to see return on investment in the short and medium term, and one of the barriers to greater investment (both public and private sector) is the lack of visible commercial and economic opportunities ahead of the long-term goal of fusion power stations. The new tech transfer hub will work with STEP, UKAEA and commercial partners to identify and then exploit tech transfer opportunities and this will remain a major focus not just for STEP but for the whole UK fusion programme for the foreseeable future. While the potential benefits of fusion energy for the environment are enormous, it will only succeed with a rigorous commercial case behind it. The UK’s approach based on collaboration between sectors, clustering and technology transfer aims to build that case robustly and provide a blueprint for commercial fusion power plants that is viable in the long term. This ethos is hardwired into every component of the UK fusion strategy, a major change over the last 10 years.

## 7. Enabling interventions

As well as specific R&D interventions, there are some areas that are uniquely for governments to address. One of these is the regulation of fusion and its application to commercial fusion ventures. The UK published a consultation paper on fusion regulation in 2021 [20], setting out options for fusion that were based on the specific hazard of fusion rather than adopting the fission regulatory approach. A subsequently published response confirmed that fusion regulation would be a matter for the Environment Agency and Health and Safety Executive in the UK rather than the Office for Nuclear Regulation. This approach it argued was proportionate to fusion hazards which are far closer to other industrial processes than existing nuclear power plants. Over-regulation of the emerging sector could stifle innovation and disincentivize investment and so the Energy Act 2023 [21] confirmed that fusion power stations would not need nuclear licensed sites. This certainty, needed to deliver STEP, is also advantageous for private sector fusion companies and in theory makes inward investment more likely. While on the surface a regulatory matter, it has strong commercial impacts and the strong signal sent to the fusion sector by the UK’s adoption of this approach is valuable for the UK. Some of the next steps for the UK include working with international partners to ensure the global approach to fusion regulation and standards is as harmonized as possible, creating a low friction global market that encourages innovation while maintaining the trust of the public. This includes providing information and expertise to the IAEA and starting to look at fusion regulation more widely. Another is publishing a ‘National Policy Statement’ on fusion power plants to help give certainty over the planning process for fusion plants, and a consultation on the NPS was published in May 2024 [22].

The second cross-cutting enabler is working with the UK skills sector and international partners to ensure that there is a sufficiently robust skills pipeline to service the growing UK fusion sector and delivery of both STEP and the wider fusion programme. This is challenging across a number of sectors, with the NSSG suggesting that the UK needs an additional 18 000 Full Time Equivalents (FTE) a year to join the nuclear industry over the next 20 years [23]. Robust analysis of the needs of the UK’s fusion sector are not yet available, but feedback received by government from companies in the fusion sector suggest that this is already starting to bite. The Fusion Futures Programme allocated over £50 million for fusion skills development. A programme of work based on the successful Oxfordshire Advanced Skills programme which works with employers, learning providers and others to deliver very high-level engineering apprenticeships, will be delivered, but expanding the focus to cover skills intervention at all career levels, including PhDs.

In summary, UK public sector activity aims to lay a stable foundation to make the UK an attractive place to undertake fusion R&D with the right proportionate level of regulation and a good supply of skills delivered in partnership with industry and employers. Alongside catalysts such as STEP providing employment opportunities in itself and in the supply chain, and an impetus for driving regulation and planning changes, there is an attractive commercial environment for fusion investment.

## 8. Conclusion

Each tenet of the UK’s fusion energy programme aims to develop a type of capability that is necessary to develop commercially viable fusion energy. Whether that is technological or scientific capability, increasing availability of skilled resource or ability to transfer technology into wider commercial markets, each part is a partnership between the public and private sector enabling those capabilities to be embedded in the organizations that will export this technology worldwide. STEP is a nexus that brings all those aspects together, tests them and is a commercial opportunity that reduces the risk for commercial and industrial organizations, enabling them to build capability. Whether STEP is ultimately the best solution for commercial fusion or not, STEP ensures that capability will exist within the UK supply chain to be deployed in fusion energy and other sectors, representing valuable economic activity of benefit to the UK economy regardless of STEP’s results. Fusion energy has potential to provide a genuinely long-term and sustainable solution to meeting growing global energy demand. While the end goal of the UK approach to fusion is to provide that solution, there are significant benefits to be had along the way. The approach adopted by the UK ensures that as far as possible, those benefits are exploited to their fullest, ensuring there is always a reason to invest in fusion energy, even before it contributes to energy generation.

## Data Availability

This article has no additional data.
